# Identification of 74 cytochrome P450 genes and co-localized cytochrome P450 genes of the CYP2K, CYP5A, and CYP46A subfamilies in the mangrove killifish *Kryptolebias marmoratus*

**DOI:** 10.1186/s12864-017-4410-2

**Published:** 2018-01-02

**Authors:** Bo-Young Lee, Duck-Hyun Kim, Hui-Su Kim, Bo-Mi Kim, Jeonghoon Han, Jae-Seong Lee

**Affiliations:** Department of Biological Science, College of Science, Sungkyunkwan University, Suwon, 16419 South Korea

**Keywords:** *Rivulus*, Killifish, Model animal, Gene family expansion, Drug metabolism, Tandem duplication

## Abstract

**Background:**

The mangrove killifish *Kryptolebias marmoratus* is the only vertebrate that reproduces by self-fertilizing and is an important model species in genetics and marine ecotoxicology. Using whole-genome and transcriptome sequences, we identified all members of the cytochrome P450 (CYP) family in this model teleost and compared them with those of other teleosts.

**Results:**

A total of 74 cytochrome P450 genes and one pseudogene were identified in *K. marmoratus*. Phylogenetic analysis indicated that the *CYP* genes in clan 2 were most expanded, while synteny analysis with other species showed orthologous relationships of CYP subfamilies among teleosts. In addition to the *CYP2K* expansions, five tandem duplicated gene copies of *CYP5A* were observed. These features were unique to *K. marmoratus*.

**Conclusions:**

These results shed a light on *CYP* gene evolution, particularly the co-localized CYP2K, CYP5A, and CYP46A subfamilies in fish. Future studies of *CYP* expression could identify specific endogenous and exogenous environmental factors that triggered the evolution of tandem *CYP* duplication in *K. marmoratus*.

**Electronic supplementary material:**

The online version of this article (10.1186/s12864-017-4410-2) contains supplementary material, which is available to authorized users.

## Background

Cytochrome P450 (CYP) enzymes are heme-containing proteins that play critical roles in the metabolism of endogenous substrates (e.g., hormones and vitamins) and in the detoxification of xenobiotics (e.g., drugs and environmental pollutants) [1–5]. Together, the CYPs constitute one of the most diverse gene families. Different species, even closely related ones, can have different numbers of *CYP* genes [6, 7]. The CYP genes are hierarchically classified at three distinct levels into subfamilies, families, and clans based on their amino acid sequence similarity, phylogenetic relationships, and syntenic relationships [6–8]. Molecular phylogenetic studies have identified ten CYP clans and 19 families in vertebrates [6, 7, 9]. CYP genes in families 1 to 4 are mainly related to xenobiotic metabolism and are more diverse than the other CYPs, with less sequence conservation [10, 11]. In contrast, CYP genes in families 5 to 51 mainly have endogenous functions. Many studies of CYP genes in families 1 to 4 have focused on ecotoxicological model species, including teleosts [1, 3, 12]. Zebrafish and Japanese medaka are the teleosts most commonly used to study the mechanistic action of CYPs in response to chemical compounds. These model organisms have shown that CYPs alert the organism to the presence of carcinogenic and hormonal disruptive substances in aquatic ecosystems [13].

Over the past two decades, CYP genes have been intensively identified and characterized in fish. More than 130 CYP genes in 19 families have been identified in all fish species examined to date [3, 10]. For instance, Japanese pufferfish (*Fugu rubripes*) have 54 *CYP* genes (later updated to 61 *CYP* genes) [8], zebrafish (*Danio rerio*) have 94 *CYP* genes (without transcript variants, the number is closer to 86) [2, 12], marine medaka (*Oryzias melastigma*) have 65 *CYP* genes [14], and channel catfish (*Ictalurus punctatus*) have 61 *CYP* genes [15]. In addition, *CYP* genes with various functions have been studied in many other fish species [1, 3, 16–19].

*Kryptolebias marmoratus* is the only vertebrate that reproduces by self-fertilization. *K. marmoratus* is a useful laboratory fish for studying molecular ecotoxicology because it is only 3–5 cm long, its life cycle is just 12–16 weeks, and it is easily maintained in aquaria [20]. As an ecotoxicological model species in which the entire genome has been sequenced [21–23], it has provided a platform for assessing the impact of various chemicals on the marine environment. In a previous study, nine *CYP* genes co-localized on a scaffold were identified and their spatio-temporal expression patterns in response to various endocrine-disrupting chemicals (EDCs) were analyzed (e.g., benzo[α]pyrene, bisphenol A, octylphenol, and nonlyphenol) [24]. In this study, we identified and annotated the full complement of 74 *CYP* genes in *K. marmoratus*. We also analyzed the co-localized CYP2K, CYP5A, and CYP46A subfamilies and characterized their structural features.

## Results

### Identification of *CYP* genes

**Fig. 1:**
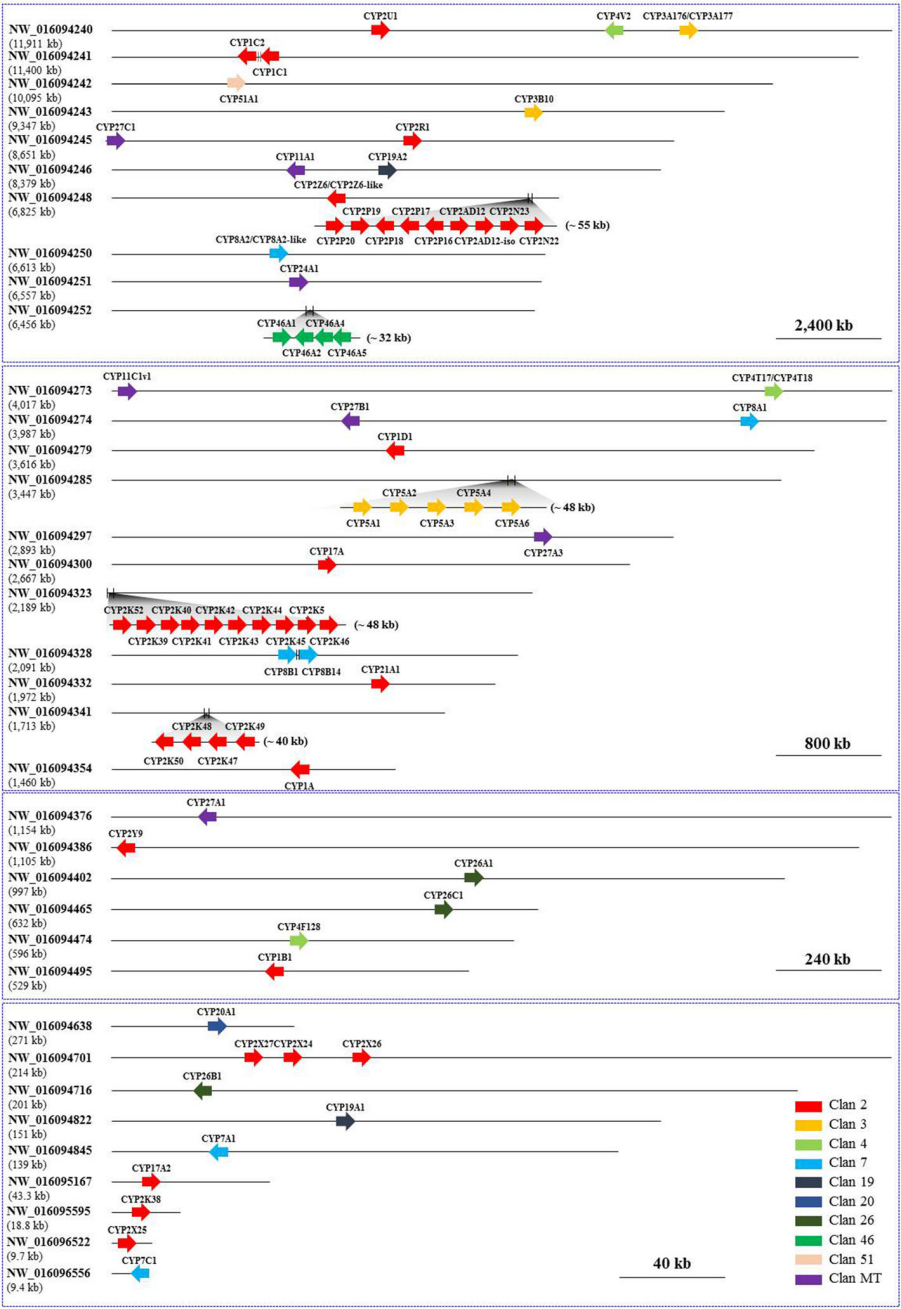
Diagram of the cytochrome P450 genes and their genomic locations in *K. marmoratus*

Using the available *K. marmoratus* genome and transcriptome assembly data, we identified 74 *CYP* genes and one *CYP* pseudogene that together mapped onto 36 scaffolds (Fig. 1; Table 1). Each scaffold contained one to ten *CYP* genes. The identified *CYP* genes were classified into ten clans (2, 3, 4, 7, 19, 20, 26, 46, 51, and mt) and 17 families (1, 2, 3, 4, 5, 7, 8, 11, 17, 19, 20, 21, 24, 26, 27, 46, and 51) (Table 1). Among the 18 teleost-specific subfamilies, *K. marmoratus* has 11 (CYP2K, CYP2N, CYP2P, CYP2V, CYP2X, CYP2Y, CYP2Z, CYP2AD, CYP3B, CYP7C, and CYP11C). Of the 74 *CYP* genes, four *CYP* genes (*CYP2Z6*, *CYP3A176*, *CYP4T17*, and *CYP8A2*) had alternatively spliced transcripts (*CYP2Z6-like, CYP3A177, CYP4T18*, and *CYP8A2-like*) (Table 1). During the *CYP* gene identification process, we obtained evidence of an additional *CYP* gene near the *CYP2K38* gene, which turned out to be a pseudogene. This pseudogene (*CYP2K38pseudo*) was discovered by mapping the *CYP2K38* gene onto the genome scaffolds. *CYP2K38pseudo* showed 98% sequence similarity in addition to structural similarity (nine exons) to *CYP2K38*, which is approximately 1 kb away on the complementary strand. However, *CYP2K38pseudo* has a stop codon at the end of the 4th exon. The corresponding transcript could not be identified from the RNA-seq data (Additional file 1: Figure S1).

**Table 1:** CYP genes identified in *K. marmoratus*

Clan	Family	CYP genes	ORF length (bp)	No. of Exons	Accession No.	Scaffold ID	Scaffold length (bp)	Start	End	Gene size (bp)	Strand
Clan 2	Family 1	CYP1A	1566	7	MF326082	NW_016094354	1,459,910	968,181	965,957	2224	–
		CYP1B1	1617	2	MF326083	NW_016094495	528,655	243,436	240,336	3100	–
		CYP1C1	1578	1	MF326084	NW_016094241	11,400,209	2,151,289	2,149,703	1586	–
		CYP1C2	1575	1	MF326085	NW_016094241	11,400,209	2,147,560	2,145,986	1574	–
		CYP1D1	1587	7	MF326086	NW_016094279	3,616,124	1,463,808	1,459,468	4340	–
	Family2	CYP2AD12	1482	9	MF326087	NW_016094248	6,824,951	6,408,131	6,411,923	3792	+
		CYP2AD12iso	1482	9	MF326088	NW_016094248	6,824,951	6,413,510	6,417,086	3576	+
		CYP2K38	1521	9	MF326089	NW_016095595	18,832	6285	9742	3457	+
		CYP2K39	1506	9	MF326090	NW_016094323	2,188,509	12,490	15,110	2620	+
		CYP2K40	1506	9	MF326091	NW_016094323	2,188,509	17,216	20,474	3258	+
		CYP2K41	1506	9	MF326092	NW_016094323	2,188,509	22,083	25,074	2991	+
		CYP2K42	1503	9	MF326093	NW_016094323	2,188,509	27,002	31,421	4419	+
		CYP2K43	1506	9	MF326094	NW_016094323	2,188,509	32,776	35,523	2747	+
		CYP2K44	1503	9	MF326095	NW_016094323	2,188,509	39,265	42,491	3226	+
		CYP2K45	1503	9	MF326096	NW_016094323	2,188,509	45,259	48,543	3284	+
		CYP2K46	1488	9	MF326097	NW_016094323	2,188,509	57,299	59,861	2562	+
		CYP2K47	1527	9	MF326098	NW_016094341	1,713,428	484,375	479,276	5099	–
		CYP2K48	1419	9	MF326099	NW_016094341	1,713,428	477,446	472,119	5327	–
		CYP2K49	1515	9	MF326100	NW_016094341	1,713,428	499,574	492,275	7299	–
		CYP2K50	1521	9	MF326101	NW_016094341	1,713,428	466,024	461,828	4196	–
		CYP2K51	1500	9	MF326102	NW_016094323	2,188,509	50,504	53,526	3022	+
		CYP2K52	1350	9	MF326103	NW_016094323	2,188,509	3986	9356	5371	+
		CYP2N22	1488	9	MF326104	NW_016094248	6,824,951	6,424,268	6,427,747	3479	+
		CYP2N23	1494	9	MF326105	NW_016094248	6,824,951	6,419,087	6,422,845	3758	+
		CYP2P16	1497	9	MF326106	NW_016094248	6,824,951	6,406,727	6,401,086	5641	–
		CYP2P17	1497	9	MF326107	NW_016094248	6,824,951	6,399,381	6,393,620	5761	–
		CYP2P18	1497	9	MF326108	NW_016094248	6,824,951	6,392,341	6,385,519	6822	–
		CYP2P19	1497	9	MF326109	NW_016094248	6,824,951	6,379,679	6,384,024	4345	+
		CYP2P20	1506	9	MF326110	NW_016094248	6,824,951	6,373,446	6,378,689	5243	+
		CYP2R1	1560	5	MF326111	NW_016094245	8,651,236	4,619,472	4,622,983	3511	+
		CYP2U1	1602	5	MF326112	NW_016094240	11,911,191	4,154,835	4,159,576	4741	+
		CYP2X24	1461	11	MF326113	NW_016094701	214,116	47,337	53,345	6008	+
		CYP2X25	1461	11	MF326114	NW_016096522	9746	N/A	5537	N/A	+
		CYP2X26	1479	11	MF326115	NW_016094701	214,116	56,638	71,729	≈ 15,091	+
		CYP2X27	1458	11	MF326116	NW_016094701	214,116	35,726	45,002	≈ 9276	+
		CYP2Y9	1476	9	MF326117	NW_016094386	1,104,698	13,051	9566	3485	–
		CYP2Z6	1515	9	MF326118	NW_016094248	6,824,951	3,433,325	3,429,696	3629	–
		CYP2Z6-like*	1500	9	MF326119	NW_016094248	6,824,951	3,433,325	3,429,696	3629	–
	Family17	CYP17A1	1548	8	MF326142	NW_016094300	2,667,381	1,094,110	1,104,539	10,429	+
		CYP17A2	1539	9	MF326143	NW_016095167	43,372	7511	12,189	4678	+
	Family21	CYP21A1	1572	12	MF326147	NW_016094332	1,971,969	1,370,794	1,374,238	3444	+
Clan3	Family3	CYP3A176	1530	13	MF326120	NW_016094240	11,911,191	8,795,434	8,800,292	4858	+
		CYP3A177*	1548	12	MF326121	NW_016094240	11,911,191	8,795,434	8,799,969	4535	+
		CYP3B10	1485	13	MF326122	NW_016094243	9,347,475	2,955,181	2,949,946	5235	–
	Family5	CYP5A1	1701	13	MF326127	NW_016094285	3,446,830	2,031,496	2,035,382	3886	+
		CYP5A2	1662	13	MF326128	NW_016094285	3,446,830	2,037,833	2,044,122	≈ 6289	–
		CYP5A3	1656	13	MF326129	NW_016094285	3,446,830	2,046,601	2,051,404	4803	+
		CYP5A4	1722	13	MF326130	NW_016094285	3,446,830	2,052,641	2,057,508	4867	+
		CYP5A6	1668	13	MF326131	NW_016094285	3,446,830	2,068,924	2,077,955	9031	+
Clan4	Family4	CYP4F128	1617	13	MF326123	NW_016094474	596,310	272,969	277,073	4104	+
		CYP4T17	1539	12	MF326124	NW_016094273	4,016,886	3,420,192	3,425,137	4945	+
		CYP4T18*	1575	10	MF326125	NW_016094273	4,016,886	3,420,192	3,425,137	4945	+
		CYP4V2	1623	11	MF326126	NW_016094240	11,911,191	7,707,782	7,701,568	6214	–
Clan7	Family7	CYP7A1	1539	8	MF326132	NW_016094845	138,999	31,992	26,587	5405	–
		CYP7C1	1563	5	MF326133	NW_016096556	9473	9282	6496	2786	–
	Family8	CYP8A1	1446	10	MF326134	NW_016094274	3,987,237	3,283,074	3,288,823	5749	+
		CYP8A2	1467	10	MF326135	NW_016094250	6,612,770	5,812,657	5,818,781	6124	+
		CYP8A2-like*	1521	10	MF326136	NW_016094250	6,612,770	5,810,209	5,818,781	8572	+
		CYP8B1	1530	1	MF326137	NW_016094328	2,090,648	988,883	990,412	1529	+
		CYP8B14	1530	1	MF326138	NW_016094328	2,090,648	995,773	997,302	1529	+
Clan19	Family19	CYP19A1	1551	9	MF326144	NW_016094822	150,677	61,672	N/A	N/A	+
		CYP19A2	1518	10	MF326145	NW_016094246	8,378,829	5,605,491	5,608,355	2864	+
Clan20	Family20	CYP20A1	1389	13	MF326146	NW_016094638	271,067	153,258	157,717	4459	+
Clan26	Family26	CYP26A1	1467	7	MF326149	NW_016094402	996,503	529,169	537,817	8648	+
		CYP26B1	1539	7	MF326150	NW_016094716	201,440	39,690	10,153	≈ 29,537	–
		CYP26C1	1608	7	MF326151	NW_016094465	632,235	493,674	502,741	9067	+
Clan46	Family46	CYP46A1	1512	15	MF326156	NW_016094252	6,456,249	3,898,087	3,893,214	4873	–
		CYP46A2	1515	15	MF326157	NW_016094252	6,456,249	3,614,329	3,621,112	6783	+
		CYP46A4	1527	15	MF326158	NW_016094252	6,456,249	3,606,320	3,612,983	6663	+
		CYP46A5	1515	15	MF326159	NW_016094252	6,456,249	3,589,333	3,605,213	15,880	+
Clan51	Family51	CYP51A1	1497	10	MF326160	NW_016094242	10,095,097	8,228,424	8,223,720	4704	–
Clanmt	Family11	CYP11A1	1572	9	MF326140	NW_016094246	8,378,829	4,196,019	4,193,094	2925	–
		CYP11C1V1	1632	9	MF326141	NW_016094273	4,016,886	9463	16,380	6917	+
	Family24	CYP24A1	1542	11	MF326148	NW_016094251	6,557,273	3,641,436	3,635,899	5537	–
	Family27	CYP27A1	1593	9	MF326152	NW_016094376	1,154,157	145,481	140,290	5191	–
		CYP27A3	1614	11	MF326153	NW_016094297	2,892,702	2,218,077	2,237,829	≈ 19,752	+
		CYP27B1	1566	9	MF326154	NW_016094274	3,987,237	1,230,560	1,227,105	3455	–
		CYP27C1	1623	9	MF326155	NW_016094245	8,651,236	41,377	52,309	≈ 10,932	+

*Alternatively spliced transcript of the gene directly above

N/A, the exact location of the gene could not be determined because the gene was mapped to the end of the scaffold

≈ Approximate genome size because the scaffold contains ‘Ns’ in the gene area

### Homology of CYP genes in other fish

**Fig. 2:**
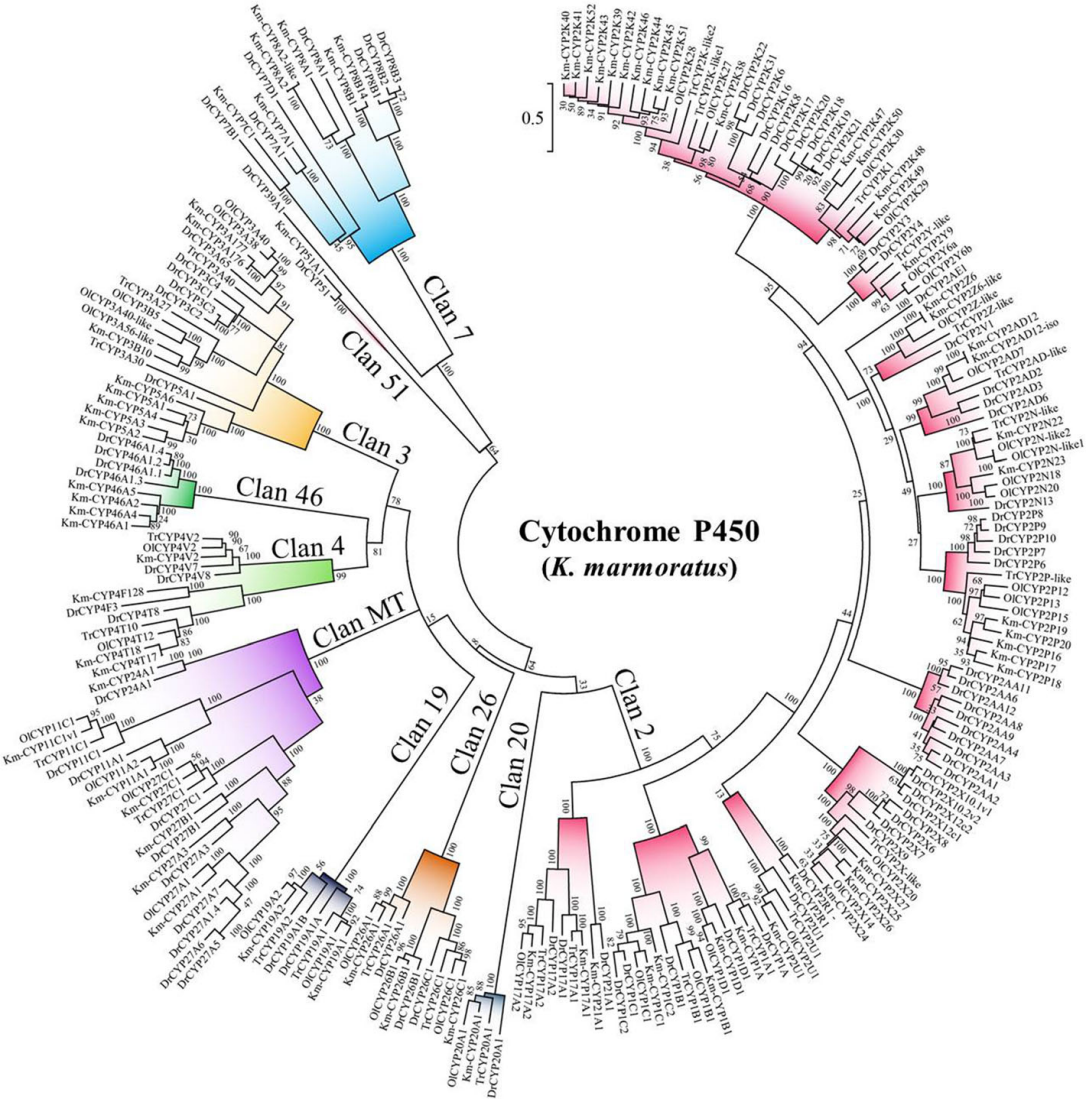
Phylogenetic tree of cytochrome P450 genes in *K. marmoratus* and other teleosts. Km, *Kryptolebias marmoratus*; Ol, *Oryzia latipes*; Dr., *Danio rerio*; Tr, *Takifugu rubripes*

**Fig. 3:**
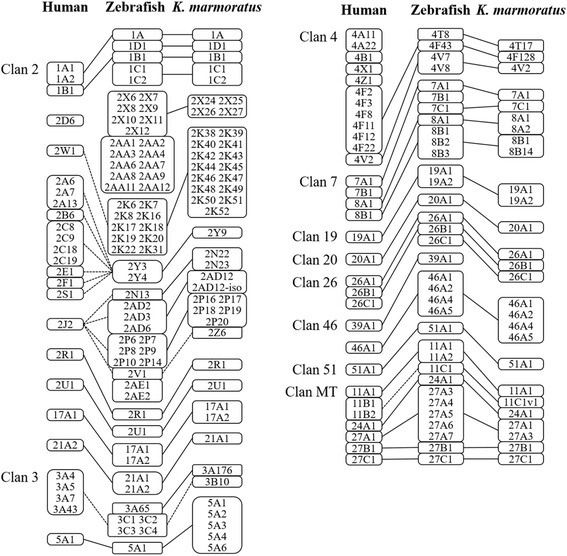
Comparison of cytochrome P450 subfamily member homologies among humans, zebrafish, and *K. marmoratus*. Image is modified from Nelson (2003)

Molecular phylogenetic analysis based on the inferred amino acid sequences was used to characterize the relationship of *K. marmoratus* CYP genes with CYP genes in other intensively studied fish species such as zebrafish (*D. rerio*), Japanese medaka (*Oryzias latipes*), and fugu (*F. rubripes*) (Fig. 2). The phylogenetic tree indicated that the clan structure was robust among these fish species with the CYP genes in clan 2 showing the most expanded pattern in *K. marmoratus* (Fig. 2). Compared with the zebrafish CYP genes, the *K. marmoratus* CYP genes were arranged into similar subfamilies, with the exception that CYP39, CYP2AA, and CYP2AE were lost in *K. marmoratus* (Fig. 3). For the CYP1, CYP17, CYP19, CYP20, CYP21, and CYP46 families, the gene members and their structures in *K. marmoratus* were similar to those in zebrafish but with different degrees of sequence similarity. Each CYP2R1 and CYP2U1 subfamily has a single *CYP* gene consisting of five exons. These genes can be considered to be orthologs of *CYP2R1* and *CYP2U1* in humans and in other fish [12, 15, 25]. CYP1A, CYP1B, CYP2U, and CYP2R appear to be evolutionarily conserved across species. In *K. marmoratus*, the CYP26 family consists of *CYP26A1, CYP26B1*, and *CYP26C1*, as shown in zebrafish. In both species, *CYP26A1* and *CYP26C1* showed similar gene structures. While zebrafish *CYP26B1* has six exons, *K. marmoratus CYP26B1* has seven exons. This difference is because the 3rd exon in zebrafish is split into two exons, thus forming the 3rd and 4th exons in *K. marmoratus*. The CYP2 family is largest in *K. marmoratus* and consists of 32 genes in nine subfamilies. The nine genes (CYP2N22, CYP2N23, CYP2AD12, CYP2AD-iso, CYP2P16, CYP2P17, CYP2P18, CYP2P19, and CYP2P20) in the three CYP2 families are homologous to human CYP2J2 because phylogenetic analysis grouped them together into a clade with the zebrafish CYP2 subfamilies (CYP2N, CYP2P, CYP2V, CYP2AD*,* and CYP2AE) (Additional file 2: Figure S2). All nine genes have been reported to be located in tandem on a scaffold (NW_016094248) and to share synteny with 11 zebrafish genes [24]. Four *CYP2X* genes are present in two separate scaffolds. The CYP2X subfamily showed a different gene structure from other members in the CYP2 family in this species with the exceptions of *CYP2R1* and *CYP2U1*. Gene members in *CYP2X* have 11 exons instead of 9 (Table 1), because the 5th and 7th exons are split into two exons each. *CYP2X25* is located on scaffold NW_016096522, while the other three *CYP2Xs* (*CYP2X27, CYP2X24,* and *CYP2X26*) are located in tandem on scaffold NW_016094701 (Fig. 1). Based on their sequence identity and the phylogenetic analysis results, we predicted that these four genes would be on the same scaffold. While the best mapping position of *CYP2X25* was on scaffold NW_016096522, the 2nd best location was the same area of *CYP2X26*. This finding is likely because the two proteins share 86% amino acid sequence similarity and the genes share 90% nucleotide sequence identity. Considering that the gaps in the area spanning the *CYPX26* gene on scaffolds NW_016094701 and NW_016096522 were relatively short, we suspected that an assembly error had occurred in the region. In order to confirm whether this was assembly errors or not, we mapped four *CYP2X* genes onto the published genome scaffolds of another killifish strain with the higher number of contigs [22]. Unfortunately, only two *CYP2X* genes (*CYP2X24* and *CYP2X25*) were mapped onto one scaffold. However, *CYP2X25*, which was isolated in this study, was mapped to one scaffold with one of four genes together and the scaffold was mapped back onto the CYP2Xs-containing scaffold (NW_016094701) of this study. Based on this analysis, this isolation of *CYP2X25* is more likely due to the assembly error, instead of the translocation.

### Tandem duplicated *CYP* genes

**Fig. 4:**
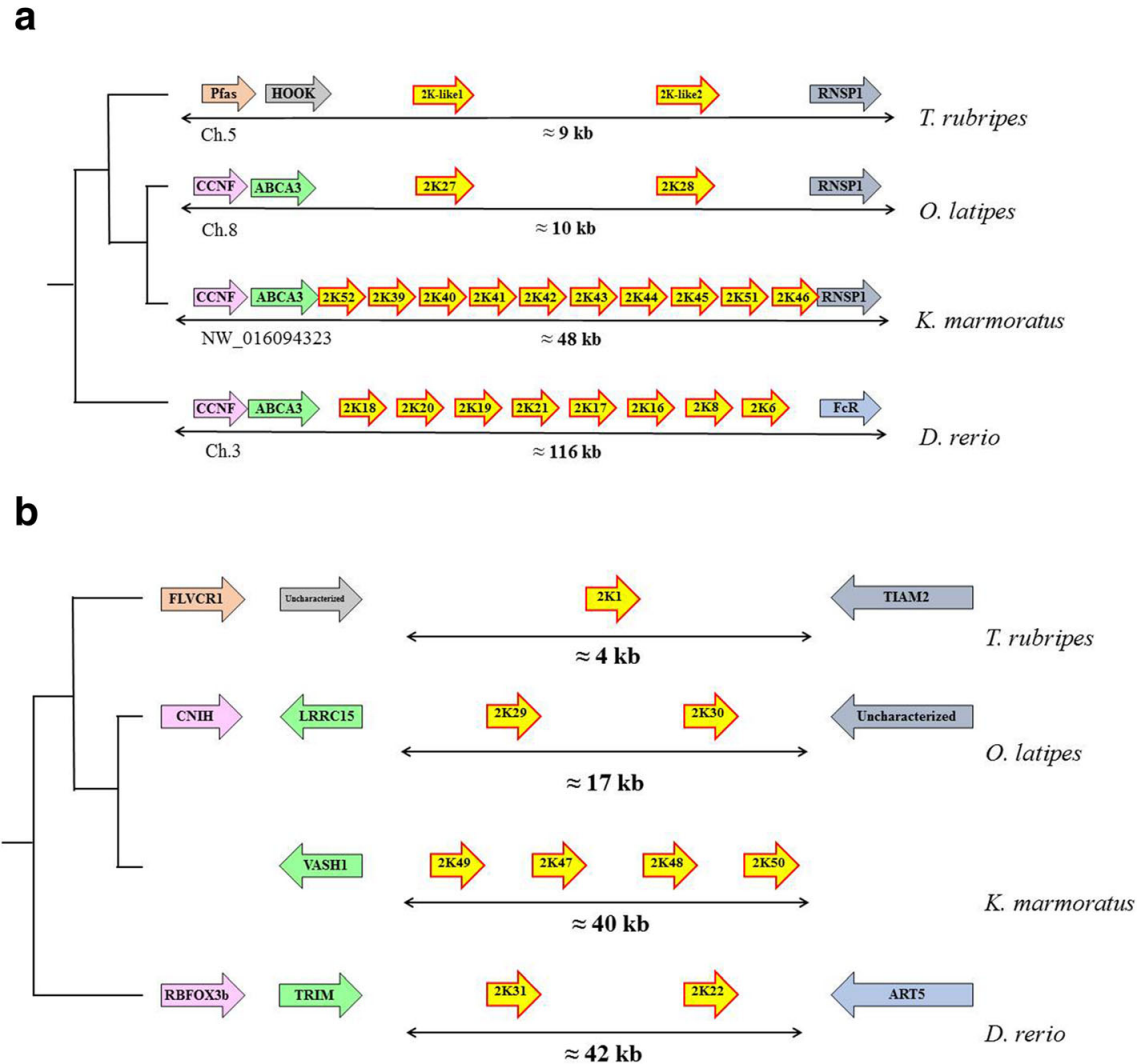
Synteny analysis of *CYP2K* genes of *K. marmoratus* and other teleosts. **a**) Synteny of the *CYP2K39–46*, *2 K51*, and *2 K52* genes. **b**) Synteny of the *CYP2K47–50* genes

**Fig. 5:**
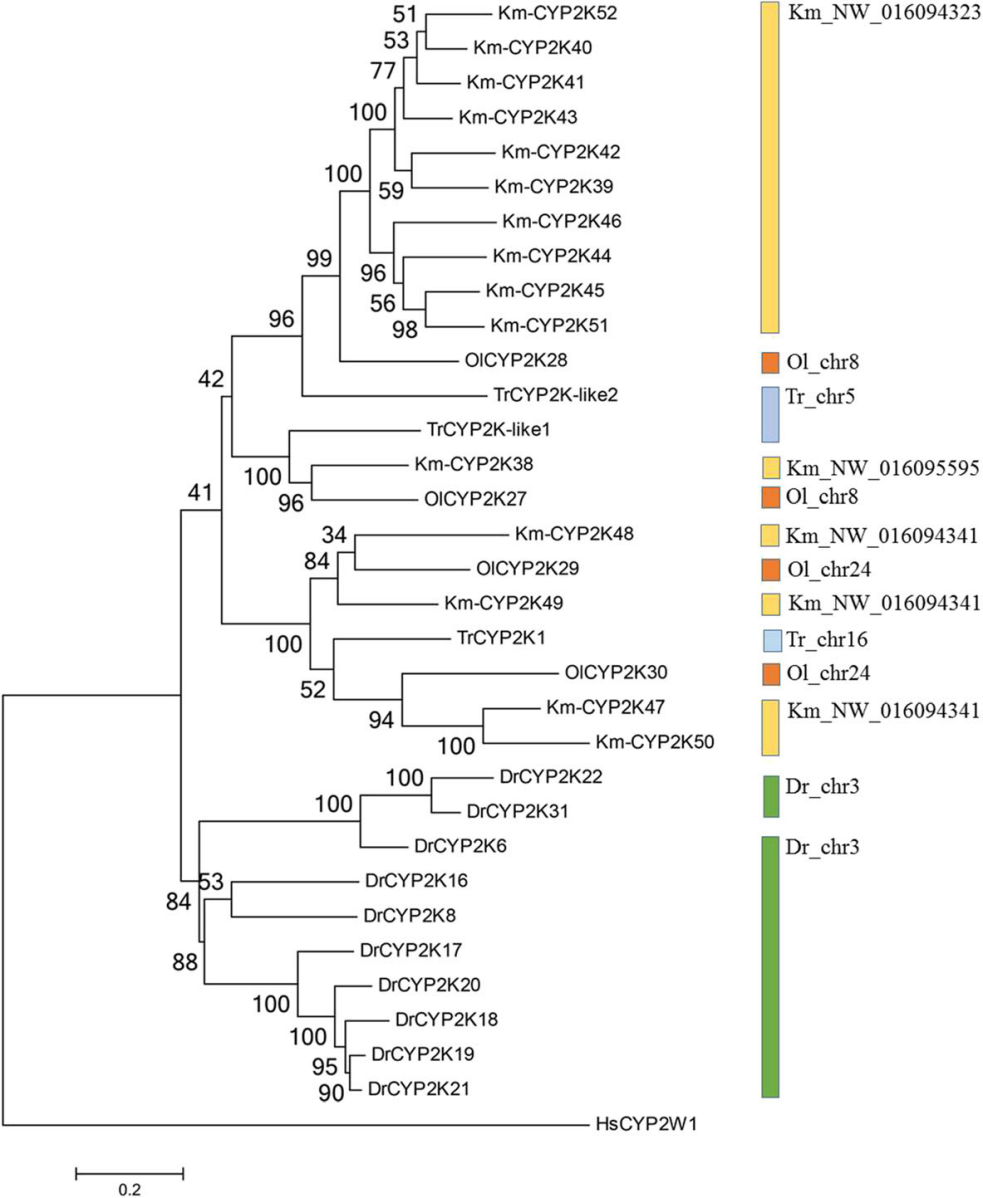
Phylogenetic tree of the CYP2K subfamily in *K. marmoratus* and other fish species with an outgroup (CYP2W1) from human. Colored bars at right side of the tree stand for grouping gene copies on particular chromosomes or scaffolds. Km, *Kryptolebias marmoratus*; Ol, *Oryzias latipes*; Dr., *Danio rerio*; Tr, *Takifugu rubripes*; Hs, *Homo sapiens*

**Fig. 6:**
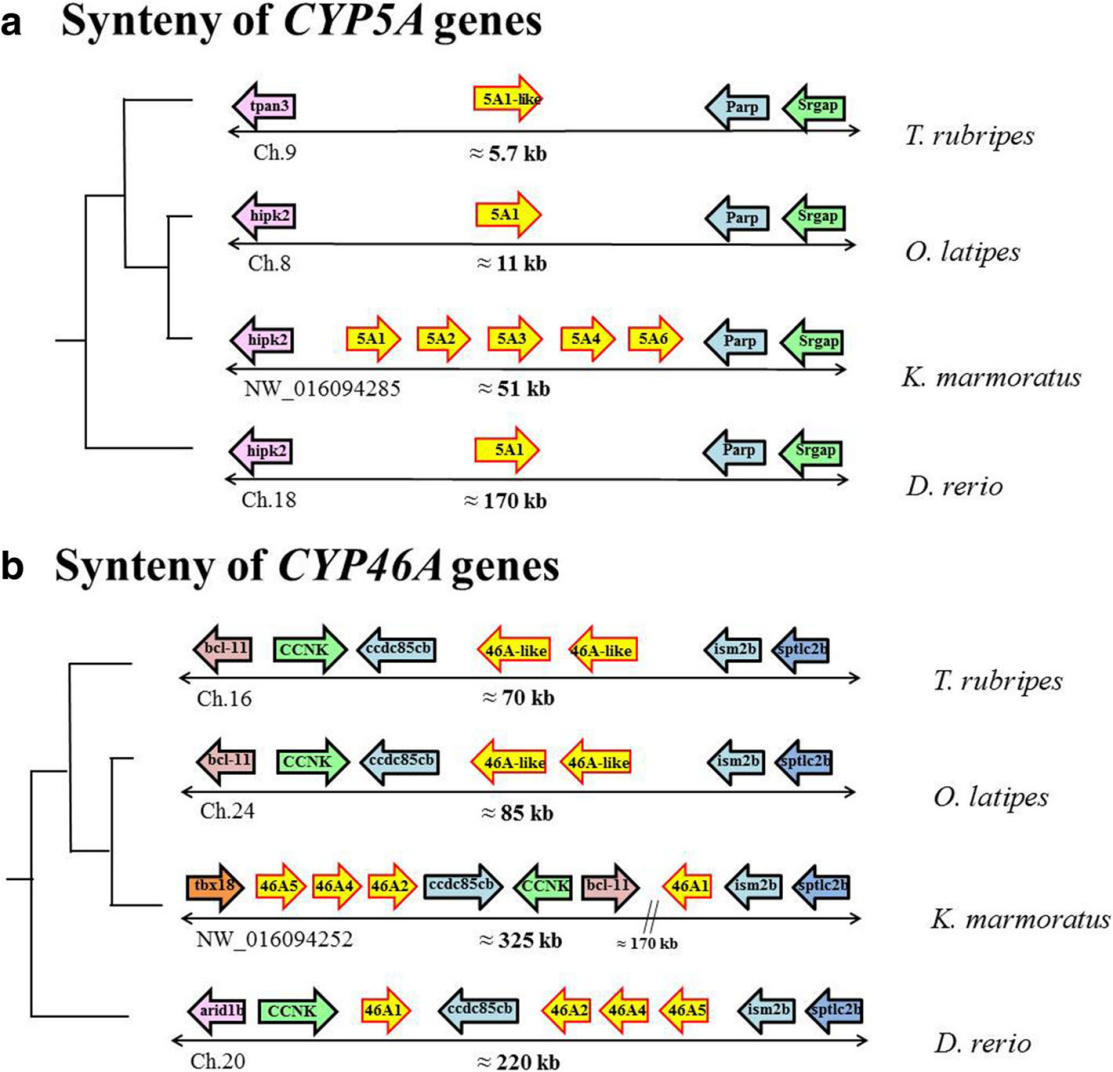
Synteny analysis of tandemly duplicated *CYP5A* (**a**) and *CYP46A* genes (**b**)

Similar to the CYP evolution patterns in other animals, tandem duplication of a number of *CYP* genes was observed in the *K. marmoratus* genome. Of 74 *CYP* genes from *K. marmoratus*, we examined the region of tandem duplicated *CYP* genes to investigate the duplicated pattern in the genome. Eight scaffolds contained more than two copies of tandem duplicated *CYP* genes, five of which had *CYP* genes with more than four copies (Fig. 1). Of CYP2K subfamily*,* ten *CYP2K* genes (*CYP2K39, CYP2K40, CYP2K41, CYP2K42, CYP2K43, CYP2K44, CYP2K45, CYP2K46 CYP2K51,* and *CYP2K52*) were clustered in the 48-kb region of scaffold NW_016094323 and four *CYP2K* genes (*CYP2K47, CYP2K48, CYP2K49,* and *CYP2K50*) were in the 40 kb region of scaffold NW_016094341 (Figs. 1 and 4). Synteny analysis revealed that zebrafish have eight *CYP2K* genes clustered in a homologous region (116 kb), whereas *T. rubripes* and *O. latipes* have only two copies of *CYP2K* genes in the 9-kb and 10-kb regions, respectively (Fig. 4a). Four *CYP2K* genes comprise another cluster on scaffold NW_016094341 (Fig. 4). Phylogenetic analysis of CYP2K genes in fish (with human genes as the outgroup) showed that the four *CYP2K* genes are similar to *medaka-CYP2KP29* and *medaka-CYP2K30*, which are located on chromosome 24 (Fig. 5). Synteny analysis of this region did not identify homologous genes outside the clusters for any species (Fig. 4). In addition, the *CYP5A* tandem genes and the *CYP46A* tandem genes were clustered in scaffolds NW_016094285 and NW_016094252, respectively (Fig. 1). While zebrafish has only one gene in the CYP5A subfamily, *K. marmoratus* has five copies of *CYP5A* genes (*5A1, 5A2, 5A3, 5A4,* and *5A6*). These copies were also arrayed in tandem on scaffold NW_016094285 (Figs. 1 and 6a). Synteny analysis showed homology with zebrafish chromosome 18 (Fig. 4b). In the CYP46A subfamily, *CYP46A1, CYP46A2, CYP46A4*, and *CYP46A5* also showed tandem duplication on scaffold NW_016094252 in the *K. marmoratus* genome (Fig. 6b). This region seemed to share synteny with *D. rerio* chromosome 20, Japanese medaka chromosome 24, and *Fugu* chromosome 16 (Fig. 6b), although some gene order mismatches in both *K. marmoratus* and *D. rerio* were observed*,* compared with pufferfish and Japanese medaka. Considering the presence of a big gap (~170 kb) between *bcl-11* and *CYP46A1* in *K. marmoratus*, we also suspected the assembly error in this region. However, comparing with the genome assembly by Kelley et al. [22], the gene order in *K. marmoratus* in both assemblies was consistent. In pufferfish and Japanese medaka, two copies of *CYP46A-like* tandem genes were surrounded by the genes, *ccdc85cb* and *ism2b*, in the synteny region. It seemed that *CYP46As* and neighboring genes, including *ccdc85cb*, *CCNK*, and *bcl*-*11*, were inverted in the area with an additional duplication of *CYP46A* copies, which was uncertain if the tandem duplication occurred before or after the inversion. Thus, based on the synteny analysis of the zebrafish, gene duplication probably has occurred prior to the inversion, although zebrafish seems to have small difference in the evolutionary repertoires in this region.

## Discussion

### Comparison of CYP subfamilies in teleosts

Using whole genome sequences and RNA-seq data, we identified a full complement of *CYP* genes in the *K. marmoratus* genome. *K. marmoratus* has a total of 74 *CYP* genes in 17 families within 10 clans. Ten clans and 19 families have been reported in vertebrate animals [6, 7, 9]. Among the 19 CYP families of vertebrates, we did not identify the CYP39 or CYP16 family in *K. marmoratus*. CYP39 families have recently been identified in teleost fish. Before this discovery, the CYP39 family was thought to be unique to mammals or to have arisen in the tetrapod lineage after it diverged from fish [8]. Goldstone et al. [12] reported the presence of *CYP39* genes in zebrafish. However, *CYP39* genes were not found in other published fish genomes, including *Fugu*. *K. marmoratus* does not have the CYP16 family. This family was lost in mammals and is also absent from zebrafish. Out of all published fish genomes, *CYP16* was reported only in *Fugu* [15].

### Gene expansion by lineage-specific duplication

While *CYP* genes are commonly expanded by tandem duplication [6, 15, 26–28], the basic mechanisms by which a certain gene is selected for such duplication remain unclear. We predominantly focused on comparing the *K. marmoratus CYP* genes with the zebrafish *CYP* genes because the two species have similar total numbers of *CYP* genes and the homology of their CYP genes with all human CYP genes is known (Fig. 3). Phylogenetic and synteny analyses revealed lineage-specific duplication of many *CYP* genes, which was apparent in some tandem duplications of *CYP* genes. Among the eight genomic regions where tandemly duplicated *CYP* genes were located in the *K. marmoratus* genome, five subfamilies (CYP2P, CYP2AD, CYP2K, CYP5A, CYP8B, and CYP46A) in the four regions showed lineage-specific duplication (Figs. 1 and 2). Although the gene members in the subfamilies were duplicated in a lineage-specific manner with different copy numbers, the syntenies (including the tandem duplicated genes) were the same between the two species (Fig. 3) [24]. Specifically, CYP46As in *K. marmoratus* and zebrafish showed strong homology within gene members and gene structures, albeit with different degrees of sequence similarity, compared to other subfamilies with the same syntenies. However, we note that gene order in the *K. marmoratus CYP46A*s synteny is different, suggesting that both species appear to have undergone evolutionary events independently after the tandem duplication of *CYP46A*. *CYP46A1* has been identified in many species, including teleosts, and plays an important role in cholesterol turnover in the central nervous system in vertebrates [29]. In humans, CYP46A1 functions as a cholesterol 24(S)-hydroxylase and a 24-hydroxy-cholesterol-hydroxylase [29–31]. Although mutations in *CYP46A1* have been associated with neurodegenerative diseases such as Alzheimer’s and Huntington’s disease in humans [32–35], the function of CYP46A1 in teleosts has not been studied. Ten *CYP2K*s on scaffold NW_016094323 belong to the subfamily that shows the highest level of lineage-specific tandem duplication in *K. marmoratus*, while four *CYP2K*s on another scaffold do not seem to be duplicated in a lineage-specific manner and share synteny with those of zebrafish (Figs. 1 and 4).

### *Kryptolebias marmoratus*-specific gene expansion

Cytochrome P450 enzymes have two main functions: metabolism of endogenous molecules and detoxification of xenobiotic compounds. Phylogenetic studies have suggested that CYP genes, which are responsible for the endogenous functions, are stable across animal species and that copy expansion is rare [11]. In contrast, CYP genes related to xenobiotic metabolism have been shown to be phylogenetically unstable with a relatively high rate of birth-death evolution [11, 36, 37]. Within this context, the most apparent gene expansion due to lineage-specific tandem duplication in *K. marmoratus* occurred in two CYP subfamilies, *CYP2K* and *CYP5A*. Similar to what has been observed in other teleost species, *CYP2K* was the most expanded subfamily in *K. marmoratus* (Fig. 4). Since *CYP2K*s are highly expanded in teleosts and the members in *CYP2K* vary across species, the functions of *CYP2K* genes have received comparatively little attention. *CYP2K*s share synteny with human *CYP2W1*, a tumor-specific CYP that oxidizes indole and chlorzoxazone [38–40]. Rainbow trout CYP2K1 and zebrafish CYP2K6 show an orthologous relationship and both metabolize aflatoxin B_1_ (AFB_1_) to exo-8,9-AFB_1_ epoxide, which is carcinogenic. However, their metabolic features differ somewhat, as only rainbow trout CYP2K1 can metabolize lauric acid [13, 41]. Based on the clan identity of CYP2K, the expansion by high level tandem duplication may have resulted from the diversity of exogenous xenobiotic substrates. Thus, rapid evolutionary selection could have favored tandem duplication as a means of coping with xenobiotic stress.

*Kryptolebias marmoratus* have five copies (*CYP5A1, CYP5A2, CYP5A3, CYP5A4*, and *CYP5A6*) of CYP5A subfamily members, while other teleosts including zebrafish, pufferfish, and channel catfish maintain the subfamily with a single gene copy [8, 12, 15]. CYP5A1 (thromboxane A2 synthase) catalyzes the conversion of prostaglandin H2 into thromboxane A2 and has been associated with human cardiovascular disease related to platelet aggregation [42]. Rather than metabolizing xenobiotics, CYP5A1 seems to be primarily involved in endogenous functions. Considering that genes involved in conserved endogenous functions are rarely expanded, the *K. marmoratus*-specific expansion of *CYP5A* is an interesting finding. Gene duplication and subsequent divergence of the duplicated copies are basic mechanisms by which gene subfamilies are formed and are considered essential sources of genetic complexity and evolutionary change [43–45]. Gene expansion by tandem duplication leading to gene clusters appears to be an important mechanism by which these needs are met for cytochrome P450 in various species. Analysis of the expression profiles of the *CYP* genes expanded specifically in *K. marmoratus* could generate insight into the endogenous and exogenous environmental factors driving CYP evolution.

## Methods

### Fish rearing

*Kryptolebias marmoratus* mangrove killifish were reared at the aquarium facility of Sungkwunkwan University (Suwon, South Korea). The fish were maintained in an automated flow-through system with constant water quality (pH 8.0 and 15 practical salinity units [psu]) at 25 °C under a 12/12-h light/dark cycle. The fish were maintained in glass aquaria (20 L capacity). Each aquarium accommodated 40 fish larvae (length ≈ 1.0 ± 0.2 cm, approximately 7 days post-hatching [dph]). Fish were fed with *Artemia* spp*.* brine shrimp (<24 h after hatching) once per day.

### Genome-wide identification of CYP genes

The assembled *K. marmoratus* whole genome (ASM164957v1) and transcriptome (SRX1765072) sequences have been published [23]. Using CYP gene sequences in other teleosts including zebrafish (*D. rerio*), Japanese medaka (*O. latipes*), and pufferfish (*F. rubripes)* (Additional file 3: Table S1), we searched for putative *CYP* sequences in the *K. marmoratus* genome. BLAST analysis of coding sequences was performed to confirm the sequence similarities. All CYP gene sequences were obtained by performing BLASTp searches of the fully assembled transcripts against the nonredundant (NR) NCBI database. A significant hit was defined as a hit with an E-value ≤10^−5^. The putative *CYP* coding sequences from *K. marmoratus* were translated into amino acids; further annotation was carried out by Prof. David R. Nelson (University of Tennessee Health Science Center) and Dr. Gared V. Goldstone (Woods Hole Oceanographic Institution). Gene structure was identified by comparing sequences between the genome scaffolds and transcriptomes. Synteny analysis was carried out by comparing the *CYP* gene clusters in *K. marmoratus* with those of Japanese medaka (*O. latipes*), pufferfish (*T. rubripes*), and zebrafish (*D. rerio*). Data were collected from the published chromosome assembly information at Ensemble (https://www.ensembl.org/index.html) with further identification.

### Phylogenetic analysis

The entire amino acid sequences encoded by the *CYP* genes of zebrafish (*D. rerio*) (Dr*-*CYPs) and Japanese medaka (*O. latipes*) (Ol-CYPs) were retrieved from GenBank (Additional file 3: Table S1). Multiple alignments of amino acid sequences from *K. mamoratus*, Japanese medaka, and zebrafish were performed with Clustal algorithm [46]. To establish a best-fit substitution model for phylogenetic analysis, the model showing the lowest score according to the Bayesian information criterion (BIC) [47] and the Akaike information criterion (AICc) [48, 49] was determined by maximum likelihood (ML) analysis. According to the results of the model test, the LG + γ + I model was chosen to generate a phylogenetic tree using MEGA6 software (Center for Evolutionary Medicine and Informatics, Tempe, AZ, USA) [50]. For phylogenetic analysis, full-length protein sequences were aligned and a phylogenetic tree was obtained as described above with an additional bootstrapping test (1000 replicates) [51]. Phylogeny data were deposited in the Treebase repository with the accession number 22004.

## Conclusions

In this study, we identified and annotated the full complement of 74 *CYP* genes in *K. marmoratus*. We also analyzed the co-localized CYP2K, CYP5A, and CYP46A subfamilies and characterized their structural features.

## Additional files

Additional file 1: Figure S1. Diagram of the process of identification of the *CYP2K38pseudo* gene. (DOC 4051 kb)

Additional file 2: Figure S2. Phylogenetic analysis of CYP genes in various fish species (marine medaka, pufferfish, stickleback, mangrove killifish) and human. (DOC 573 kb)

Additional file 3: Table S1. Accession numbers of genes used for synteny and phylogenetic analysis. (DOCX 19 kb)

## Abbreviations

CYP: Cytochrome P450
