# Supplementary material for: Identification of 74 cytochrome P450 genes and co-localized cytochrome P450 genes of the CYP2K, CYP5A, and CYP46A subfamilies in the mangrove killifish Kryptolebias marmoratus
Source: BMC Genomics. 2018 Jan 2;19:7. doi: 10.1186/s12864-017-4410-2 (PMC5751882; doi:10.1186/s12864-017-4410-2)
Supplement: Supplementary file 3 — Accession numbers of genes used for synteny and phylogenetic analysis. (DOCX 19 kb) [file 12864_2017_4410_MOESM3_ESM.docx]

**Suppl. Table 1**. Accession numbers of genes used for synteny and phylogenetic analysis

| **Gene name** | **Accession No.** | **Gene name** | **Accession No.** | **Gene name** | **Accession No.** |
| --- | --- | --- | --- | --- | --- |
| DrCYP1A | NM_131879.2 | DrCYP17A2 | NM_001105670.1 | TrCYP4V2 | XM_003972307.2 |
| DrCYP1B1 | NM_001045256.1 | DrCYP19A1A | NM_131154.3 | TrCYP11C1 | XM_003977621.1 |
| DrCYP1C1 | NM_001020610.2 | DrCYP19A1B | NM_131642.2 | TrCYP20A1 | XM_003966666.2 |
| DrCYP1C2 | NM_001114849.1 | DrCYP20A1 | NM_213332.2 | TrCYP26A1 | XM_003977972.2 |
| DrCYP1D1 | NM_001007310.1 | DrCYP24A1 | NM_001089458.1 | TrCYP26C1 | XM_011603005.1 |
| DrCYP2AA1 | NM_001025557.2 | DrCYP26A1 | NM_131146.2 | TrCYP27C1 | XM_011610083.1 |
| DrCYP2AA3 | NM_198369.2 | DrCYP26B1 | NM_212666.1 | TrCYP17A1 | NM_001105236.1 |
| DrCYP2AA4 | NM_001002092.2 | DrCYP26C1 | NM_001029951.2 | TrCYP19A1 | NM_001280028.1 |
| DrCYP2AA6 | NM_001199969.3 | DrCYP27A1.4 | NM_001328513.1 | TrCYP17A2 | EF624005.1 |
| DrCYP2AA7 | NM_001109722.1 | DrCYP27B1 | NM_001311791.1 | TrCYP19A2 | NM_001173496.1 |
| DrCYP2AA8 | NM_001006080.1 | DrCYP27C1 | NM_001113337.2 | TrCYP2AD-like | XR_965373.1 |
| DrCYP2AA9 | NM_001079851.1 | DrCYP39A1 | NM_001030189.2 | TrCYP2K-like2 | XM_003979997.1 |
| DrCYP2AA12 | NM_001109701.2 | DrCYP46A1.1 | NM_001020522.1 | OlCYP11C1 | AB105880.1 |
| DrCYP2AD2 | NM_152954.1 | DrCYP46A1.2 | NM_200461.1 | OlCYP1C1 | EF546468.1 |
| DrCYP2AD3 | NM_001025554.2 | DrCYP46A1.3 | NM_001045298.1 | OlCYP11A2 | NM_001163086.1 |
| DrCYP2AD6 | NM_001082936.1 | DrCYP46A1.4 | NM_200479.2 | OlCYP2P13 | NM_001163163.1 |
| DrCYP2K16 | NM_001005963.2 | DrCYP5A1 | NM_205609.2 | OlCYP3A40 | NM_001105095.1 |
| DrCYP2K17 | NM_001042778.2 | DrCYP51 | NM_001001730.2 | OlCYP3A38 | AF105018.1 |
| DrCYP2K6 | NM_200509.1 | DrCYP2K31 | XM_001334054.3 | OlCYP19A1 | NM_001278879.1 |
| DrCYP2K8 | NM_001130623.2 | DrCYP2AA2 | NM_001077458.1 | OlCYP26A1 | EF535796.1 |
| DrCYP2K18 | NM_200512.1 | DrCYP11C1 | NM_001080204.1 | OlCYP26B1 | EF535797.1 |
| DrCYP2K19 | NM_001079704.2 | DrCYP2X12c2 | XM_005166521.4 | OlCYP17A2 | EF423918.1 |
| DrCYP2K22 | NM_200235.1 | DrCYP2X12c1 | NM_001079853.1 | OlCYP1D1 | XM_020706069.1 |
| DrCYP2N13 | NM_001007356.1 | DrCYP2Y4 | XM_017351113.2 | OlCYP1B1 | XM_020709261.1 |
| DrCYP2P6 | NM_200139.1 | DrCYP3C2 | XM_021474709.1 | OlCYP2Y6b | XM_004075220.3 |
| DrCYP2P7 | NM_001083049.1 | DrCYP7D1 | XM_682404.6 | OlCYP2X14 | XM_004069766.3 |
| DrCYP2P8 | NM_001083035.2 | DrCYP27A3 | NM_001123277.1 | OlCYP2Y6a | XM_004075218.3 |
| DrCYP2P9 | NM_200620.1 | DrCYP27A6 | NM_001045304.1 | OlCYP2P12 | XM_011474367.2 |
| DrCYP2P10 | NM_201511.1 | DrCYP2R1 | XM_686732.7 | OlCYP2AD7 | XM_004068191.3 |
| DrCYP2U1 | NM_001145564.1 | DrCYP7B1 | XM_693936.8 | OlCYP2P15 | XM_004068192.3 |
| DrCYP2V1 | NM_001009890.2 | DrCYP21A1 | XM_021474355.1 | OlCYP2N20 | XM_004068186.3 |
| DrCYP2X6 | NM_001013565.1 | DrCYP27A7 | XM_002663399.5 | OlCYP2Z5 | XM_020702698.1 |
| DrCYP2X7 | NM_001256177.1 | DrCYP2AA11 | XM_017353668.2 | OlCYP2N18 | XM_004068188.3 |
| DrCYP2X8 | NM_001002187.1 | DrCYP2AE1 | XM_021470048.1 | OlCYP2X20 | XM_004085472.3 |
| DrCYP2X9 | NM_001077453.1 | DrCYP27A5 | XM_009304845.3 | OlCYP2K30 | XM_004083796.2 |
| DrCYP2X10.1v1 | NM_001044309.2 | DrCYP2K20 | XM_021473128.1 | OlCYP2K27 | XM_004071425.3 |
| DrCYP2X10.2v2 | NM_001328515.1 | DrCYP2K21 | XM_687463.8 | OlCYP2K29 | XM_004083641.3 |
| DrCYP2Y3 | NM_001020822.1 | DrCYP8A1 | NM_001111160.1 | OlCYP2K28 | XM_011478173.2 |
| DrCYP3A65 | NM_001037438.1 | TrCYP1A1 | XM_003978785.2 | OlCYP2U1 | XM_020703523.1 |
| DrCYP3C1 | NM_212673.1 | TrCYP1B1 | XM_003978309.2 | OlCYP3B5 | XM_011484276.2 |
| DrCYP3C3 | NM_001007400.2 | TrCYP2Y-like | XM_003968682.2 | OlCYP3A40-like | XM_011484277.2 |
| DrCYP3C4 | NM_001077548.1 | TrCYP2X-like | XM_003977732.2 | OlCYP3A56-like | XM_004077282.3 |
| DrCYP4F3 | NM_001089541.1 | TrCYP2P-like | XM_003974024.2 | OlCYP4T12 | XM_004078270.3 |
| DrCYP4T8 | NM_199216.1 | TrCYP2N-like | XM_003974025.2 | OlCYP4V2 | XM_004065956.3 |
| DrCYP4V7 | NM_001079996.1 | TrCYP2Z-like | XM_003974039.2 | OlCYP20A1 | XM_004066358.3 |
| DrCYP4V8 | NM_001077602.1 | TrCYP2K1 | XM_003971443.2 | OlCYP26C1 | XM_004076978.3 |
| DrCYP7A1 | NM_201173.2 | TrCYP2K-like1 | XM_003964646.2 | OlCYP27A1 | XM_004081817.3 |
| DrCYP8B1 | NM_001003736.1 | TrCYP2U1 | XM_011612899.1 | OlCYP27C1 | XM_004067082.3 |
| DrCYP8B2 | NM_001110288.1 | TrCYP3A27 | XM_003963871.2 | OlCYP2N-like1 | XM_004068280.3 |
| DrCYP8B3 | NM_001004587.2 | TrCYP3A30 | XM_011612794.1 | OlCYP2N-like2 | HF933210.1 |
| DrCYP11A1 | NM_152953.2 | TrCYP3A40 | XM_003963277.2 | OlCYP19A2 | NM_001105093.2 |
| DrCYP17A1 | NM_212806.3 | TrCYP4T10 | XM_011618192.1 |  |  |
