# Supplementary material for: Identification of 74 cytochrome P450 genes and co-localized cytochrome P450 genes of the CYP2K, CYP5A, and CYP46A subfamilies in the mangrove killifish Kryptolebias marmoratus
Source: BMC Genomics. 2018 Jan 2;19:7. doi: 10.1186/s12864-017-4410-2 (PMC5751882; doi:10.1186/s12864-017-4410-2)
Supplement: Supplementary file 2 [file 12864_2017_4410_MOESM1_ESM.doc]

Suppl. Fig. 1

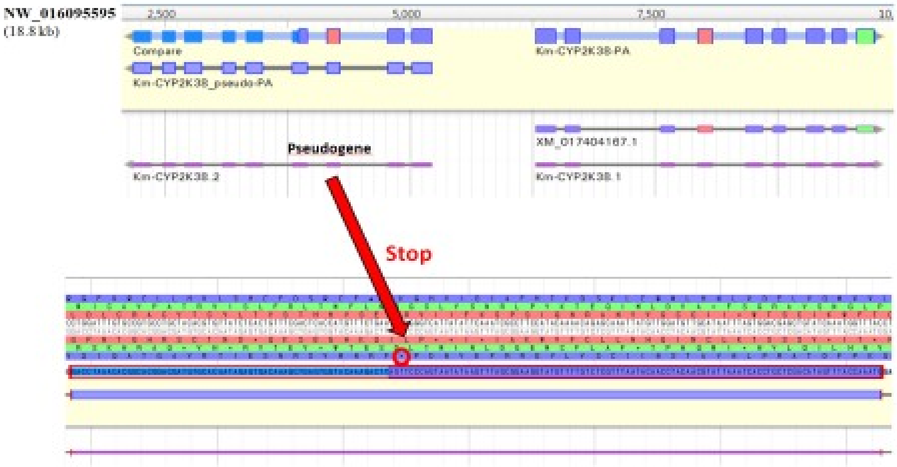

NW_016095595
(18.8 kb)
2,500
5,000
7,500
Compare
Km-CYP2K38-PA
Km-CYP2K38_pseudo-PA
XM_017404167.1
Pseudogene
Km-CYP2K38.2
Km-CYP2K38.1
Stop
