# Supplementary material for: Identification of 74 cytochrome P450 genes and co-localized cytochrome P450 genes of the CYP2K, CYP5A, and CYP46A subfamilies in the mangrove killifish Kryptolebias marmoratus
Source: BMC Genomics. 2018 Jan 2;19:7. doi: 10.1186/s12864-017-4410-2 (PMC5751882; doi:10.1186/s12864-017-4410-2)

Suppl. Fig. 2

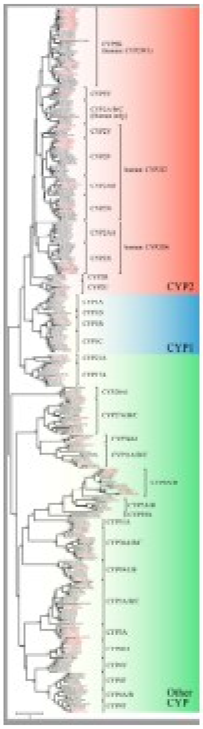

Supplement: Supplementary file 3 [file 12864_2017_4410_MOESM2_ESM.doc]
